# Mind-Wandering Mediates the Associations Between Neuroticism and Conscientiousness, and Tendencies Towards Smartphone Use Disorder

**DOI:** 10.3389/fpsyg.2021.661541

**Published:** 2021-08-30

**Authors:** Marko Müller, Cornelia Sindermann, Dmitri Rozgonjuk, Christian Montag

**Affiliations:** ^1^Department of Molecular Psychology, Institute of Psychology and Education, Ulm University, Ulm, Germany; ^2^Institute of Mathematics and Statistics, University of Tartu, Tartu, Estonia

**Keywords:** mind-wandering, fear of missing out, big five personality traits, neuroticism, conscientiousness, smartphone overuse, smartphone use disorder, structural equation modeling

## Abstract

Mounting evidence suggests that smartphone overuse/*sm*artphone *u*se *d*isorder (SmUD) is associated with negative affectivity. Given a large number of smartphone users worldwide (currently about 4.7 billion) and the fact that many individuals carry their smartphones around 24/7, it is of high importance to better understand the phenomenon of smartphone overuse. Based on the *i*nteraction of *p*erson-*a*ffect-*c*ognition-*e*xecution (I-PACE) model, we investigated the links between SmUD and the personality traits, neuroticism and conscientiousness, which represent two vulnerability factors robustly linked to SmUD according to a recent meta-analysis. Beyond that, we tested the effects of *m*ind-*w*andering (MW) and *f*ear *o*f *m*issing *o*ut (FoMO) in the relation between individual differences in personality and tendencies towards SmUD. The effective sample comprised 414 study participants (151 men and 263 women, age *M* = 33.6, *SD* = 13.5). By applying a *s*tructural *e*quation *m*odeling (SEM) technique, we observed that the associations of higher neuroticism and lower conscientiousness with higher levels of SmUD were mediated by higher scores in mind-wandering. These novel findings can help to understand the associations between personality and SmUD in more detail.

## Introduction

### Smartphone Use and Smartphone Use Disorder

Currently, there are 4.7 billion smartphone users worldwide (Statista, 2021), and this technology may spread around the globe faster than any other we have seen so far (DeGusta, 2012). Without doubt, this mobile device provides a variety of advantages, such as the opportunity to stay in touch with beloved ones via far distances. It can also increase productivity (Bertschek and Niebel, 2016; Lee et al., 2017), for instance, by allowing for more efficient navigation in unknown territory. On the other hand, mounting evidence suggests that smartphone use can also be harmful, which is currently being intensely investigated and debated among researchers. One of the downsides of the increasing incorporation of smartphones in everyday life is the phenomenon of smartphone overuse. A broader literature overview on the detrimental effects including social, psychological and physical problems, next to comorbidities as well as mental health issues (such as stress, depression, and anxiety) examined in the realm of smartphone overuse is provided in the works of Gutiérrez et al. (2016) and Elhai et al. (2019).

Commonly used measurement tools for smartphone overuse originate from traditional substance-abuse criteria [for a description of different tools, see Billieux (2012)], as we did in this study, for reasons of comparability (Kwon et al., 2013). This is why smartphone overuse is often denoted as “smartphone addiction” (Ting and Chen, 2020). However, researchers still lack consensus on a conceptual definition of smartphone overuse (Billieux et al., 2015; Panova and Carbonell, 2018). This is also reflected by the variety of existing terms in the literature to capture this phenomenon arising from the field of “cyber addictions” (Suissa, 2014) or “technological addictions” (Griffiths, 1995; Carbonell et al., 2016; Kuss and Billieux, 2017). Some adjectives used to describe smartphone overuse include “compulsive,” “pathological,” “excessive,” “dysfunctional,” and “problematic” (Billieux, 2012; Elhai et al., 2017; van Velthoven et al., 2018). For consistency, we will use the term *sm*artphone *u*se *d*isorder (SmUD), as discussed in Montag et al. (2021).

A recent taxonomy introduced in the review article just mentioned stresses the relevance to distinguish between non-mobile and mobile versions of *I*nternet-*u*se *d*isorder (IUD); SmUD can be characterized as a mobile version of IUD (Montag et al., 2021). By being constantly connected to the Internet, the smartphone provides users with ubiquitous access to the online world as long as there is reception. According to current conceptualizations, there are generalized (non-specific) and specific IUDs. Generalized (non-specific) IUD deals with the general overuse of the Internet (e.g., browsing or aimlessly surfing the Internet). Specific IUDs deal with the overuse of specific online activities comprising communication, pornography, buying-shopping, gaming, and gambling via a device of choice [for empirical studies, see also Montag et al. (2015a), Müller et al. (2017)]. Importantly, neither generalized IUD, nor SmUD or most of the specific IUDs are currently included in official diagnostic manuals. Only gaming and gambling disorders (both predominantly online and offline) have been recognized as mental health disorders in the 11th Revision of the *I*nternational *C*lassification of *D*iseases (ICD-11) by the *W*orld *H*ealth *O*rganization (WHO, 2021). Of importance for the present work, these two recently accepted disorders belong to the category of addictive behaviors in ICD-11, a currently discussed and intensely studied area that also touches the overuse of the smartphone (or some of its apps/contents). Although not an official diagnosis, SmUD tendencies are being investigated by many researchers around the world.

### Theoretical Conceptualization of the Emergence and Maintenance of Smartphone Use Disorder

The theoretical framework to conceptualize the current study is called the I-PACE (*i*nteraction of *p*erson-*a*ffect-*c*ognition-*e*xecution) model by Brand et al. (2016). It was initially established to explain the emergence of specific IUDs, but is commonly used in research focusing on SmUD and its interplay with psychological and behavioral factors [e.g., see works by Elhai et al. (2020c), Elhai et al. (2019)].

According to the I-PACE model, interactions (I) of a person's (P) core characteristics (e.g., personality traits, genetics, psychopathology, social cognition) and behavior-specific predisposing factors (e.g., needs, motives and values for going online) influence one's perception of external and internal (mood modification) triggers, and, in turn, the reaction in personal stressful or critical situations. Individual coping styles and reward expectancies may lead to affective (A) and cognitive (C) responses, such as salience that can induce a desire or strong need to use certain Internet activities (e.g., social media or online gaming apps). In other words, increased attention to stimuli as well as longing for a particular action may result in succumbing to the craving due to impairments in executive functioning (E), such as inhibitory control and decision making (Brand et al., 2016), to receive the desired gratification (Blumler, 1979; Sundar and Limperos, 2013). Based on the I-PACE model, stable personality traits (P) can be seen as predisposing variables contributing to both the emergence and the maintenance of SmUD. Similarly, and in line with this model, FoMO and *m*ind-*w*andering (MW) might be seen as affective (A) and cognitive (C) variables mediating the relation between personality and SmUD (Brand et al., 2016, 2019).

Another important theory to be named in the context of our work explicitly targets the smartphone. Billieux's model (2012) specifically highlights the role of personality to better understand smartphone overuse and foresees neuroticism as a prominent risk factor. This assumption has been backed up in a recent meta-analysis showing robust links between higher neuroticism/lower conscientiousness and SmUD (Marengo et al., 2020).

### Personality Predispositions of SmUD

Given the results from the aforementioned meta-analysis by Marengo et al. (2020), the associations of both higher neuroticism and lower conscientiousness with higher levels of SmUD can be considered robust. However, it is less clear which cognitive and affective processes meditate the links between personal characteristics and SmUD. Therefore, the present work aims not only to replicate the above associations between personality traits and SmUD, but also to investigate the potential underlying mechanisms by including FoMO and MW as mediating variables. Both are of great interest, because they have been associated with other key variables of the present work: SmUD, neuroticism, and conscientiousness (see in the following sections “FoMO as Mediating Variable in the Relations Between the Big Five and SmUD” and “Mind-Wandering as a Mediator in the Big Five-SmUD Association” for a more detailed elaboration).

### FoMO as Mediating Variable in the Relations Between the Big Five and SmUD

FoMO describes the permanent concern that others (e.g., one's friends) have fulfilling experiences excluding oneself and the wish to stay connected with peers. The role of social media's design in triggering FoMO is at least theoretically well-established (Alutaybi et al., 2019; Montag et al., 2019), and research has shown that FoMO is robustly linked to SmUD (Chotpitayasunondh and Douglas, 2016; Elhai et al., 2016, 2018, 2020b,c,d; Fuster et al., 2017; Kuss and Griffiths, 2017; Oberst et al., 2017; Gezgin, 2018; Liu and Ma, 2018; Wolniewicz et al., 2018; Sha et al., 2019).

Moreover, FoMO shows associations with personality variables. In particular, it is negatively linked to conscientiousness and positively to neuroticism (Stead and Bibby, 2017; Rozgonjuk et al., 2021). In parallel with the strong connection of FoMO and neuroticism, its association with negative affectivity (Elhai et al., 2018, 2020a) underscores that FoMO can be conceptualized as an affective and cognitive response in light of the I-PACE model. Against this background, FoMO is also understood as a specific cognition process (Balta et al., 2020) that represents a maladaptive cognitive coping strategy (Elhai et al., 2019) or a cognitive bias (Wegmann et al., 2017; Elhai et al., 2020c) mediating the relationship between individual's core characteristics, such as neuroticism, and SmUD (Balta et al., 2020). In addition to FoMO's negative associations with emotional stability (inverse of neuroticism) and conscientiousness, Stead and Bibby (2017) verified a profound positive link between FoMO and problematic internet use—focusing on social media use. In the present study, FoMO is modeled as a mediator in the link between personality traits (neuroticism and conscientiousness) and SmUD.

### Mind-Wandering as a Mediator in the Big Five-SmUD Association

Next to FoMO, mind-wandering is of importance for the present study. It can broadly be defined as thought leaps not related to the currently executed task (Mrazek et al., 2013b). MW, thus, is closely related to inattention. This is also reflected in the items of the instrument used in the present study to measure levels of mind-wandering, the *M*ind-*W*andering *Q*uestionnaire (MWQ) by Mrazek et al. (2013b). Despite the relatedness of mind-wandering and inattention, the naming of the applied questionnaire (MWQ), and for the sake of clarity, we decided to use the term “mind-wandering” throughout the whole study. With the MWQ, trait levels of the regular occurrence of mind-wandering can be investigated as done by Mrazek et al. (2013b).

Links between mind-wandering and personality traits, including low conscientiousness and high neuroticism, have been demonstrated using different instruments^1^ (Giambra, 1980; Carciofo et al., 2016). Further works, also applying various measures, underpin the link of mind-wandering with the Big Five (neuroticism and conscientiousness) (Jackson and Balota, 2012,^2^; Robison et al., 2017,^3^; Vannucci and Chiorri, 2018,^4^). In a related field of research, Nigg et al. (2002) discovered that higher attention problems (domain of the Wender-Stein ADHD Scales) were linked to lower conscientiousness and moderately correlated with higher neuroticism. The diversity of mind-wandering measures once again shows how intertwined the construct is with related scientific disciplines, especially the field of attention/inattention.

Very little about the direct connections between mind-wandering itself and SmUD can be found in the literature. However, we identified a few studies pointing at a putative link between both aforementioned constructs: Mind-wandering on a daily basis (measured by four different instruments^5^) was positively associated with general smartphone use (sending and receiving texts, using social media, reading news, etc.) and above all with absent-minded smartphone use. The latter clearly dominates the connection with mind-wandering (Marty-Dugas et al., 2018). Furthermore, a significant relationship between inattentive behavior^6^ and SmUD has been discovered in the related research area mentioned above (Kim et al., 2020).

### FoMO and Mind-Wandering

Little research has investigated FoMO's link with mind-wandering. While the primary focus has been on examining the impact of smartphone notifications (Fitz et al., 2019)^7^, no study has assessed the direct association between FoMO and mind-wandering. FoMO has been linked to disrupted activities due to interruptive notifications (Rozgonjuk et al., 2019). Moreover, switching off notifications was found to trigger anxiety (Kushlev et al., 2016) and, people were afraid to miss out important messages on their mobile phones (Pielot and Rello, 2015). With regard to mind-wandering, Kushlev et al. (2016) found a positive association of push-notifications with inattention (which is conceptionally similar to mind-wandering; see section “Mind-Wandering as a Mediator in the Big Five-SmUD Association”). However, no literature has been found that investigated the direct link between the two constructs.

### Aims and Hypotheses of the Present Study

In this work, we want to shed more light on the hypothesized mechanisms underlying the associations between personality (P) and SmUD using a *s*tructural *e*quation *m*odeling (SEM) technique. Based on Billieux's *relationship maintenance pathway* and the I-PACE framework, we expect FoMO and mind-wandering—representing affective (A) and cognitive (C) processes—to mediate the relationship between personality, specifically neuroticism and conscientiousness, towards SmUD. Given the scarce literature on associations between FoMO and mind-wandering, we did not pose a hypothesis, and the investigation of this link was rather explorative.

Although, the bivariate associations between personality, FoMO, mind-wandering, and SmUD have found substantial support in previous works. Research bringing all these variables together in a coherent model, to our knowledge, is non-existent. This makes the present study a novel contribution to the field of research on the underlying mechanisms of SmUD.

**Figure 1:**
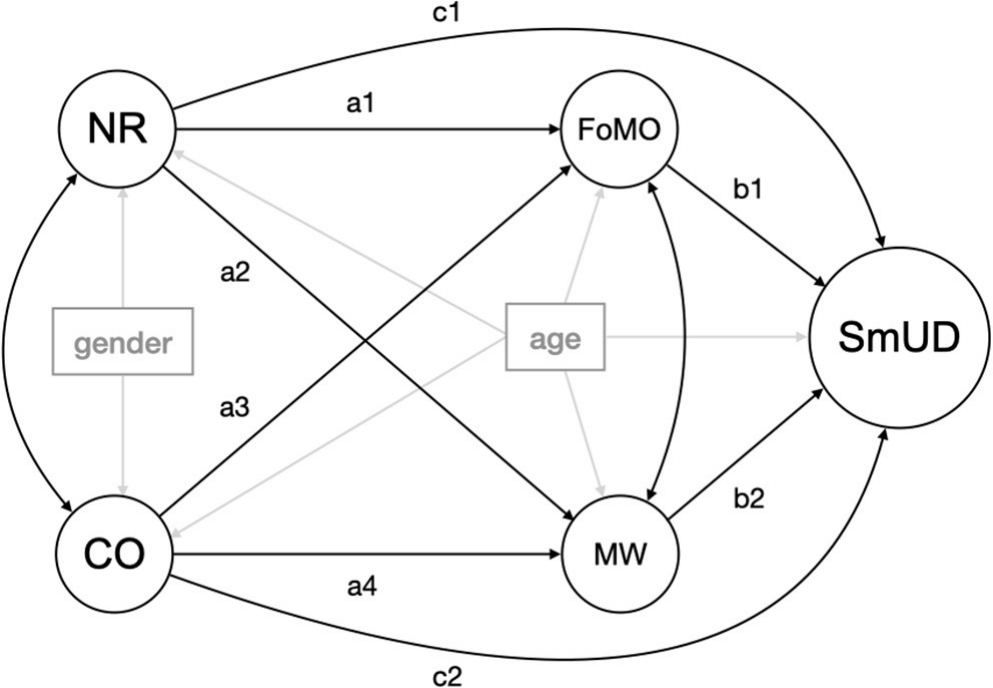
Graphical representation of the theoretical research model. It shows the assembly of the latent constructs, depicted as circles (NR, CO, FoMO, MW, SmUD) and measured covariates (age, gender) displayed as rectangles (manifest variables). For reasons of clarity, items of latent variables are not presented here, however, they were used in calculating the model. Paths illustrated with one arrow tip signify a regression effect on the construct they point at, and two arrow tips indicate a covariance between two constructs. CO, Conscientiousness; NR, Neuroticism; MW, Mind-Wandering; FoMO, Fear of Missing Out; SmUD, Smartphone Use Disorder. We also note that the covariates age/gender have often been associated with several of the investigated variables (for reasons of brevity not discussed in this work) and, therefore, need to be controlled for.

Our model is visualized in Figure 1. Based on the body of literature cited above, we test the following hypotheses:

H1: Higher neuroticism and lower conscientiousness are associated with higher SmUD.
H2: Higher neuroticism is associated with higher FoMO and higher mind-wandering levels.
H3: Lower conscientiousness is associated with higher FoMO and higher mind-wandering levels.
H4: The associations of neuroticism and conscientiousness with SmUD are mediated by FoMO and mind-wandering.

## Materials and Methods

### Procedure

Anyone, who (i) was at least 12 years old, (ii) had Internet access, and (iii) understood the questions asked in German was allowed to participate. Prior to taking part in the study, all participants were informed about the procedures, and they gave their electronic consent for participation. If the participants were underaged (at least 12, but under 18 years old), parental or legal guardian electronic informed consent needed to be provided. Data of the convenience sample were collected from January to August 2020 from German-speaking smartphone users [on the Surveycoder platform, developed by Kannen (2018)]. The study was advertised on German television, in print media, and on the Internet. To attract people to take part, the participants received immediate feedback about their personality, smartphone and social media use after completing the survey. The entire online study was administered in German language. We collected data on basic sociodemographic variables, such as age, gender, country of residence, and the highest level of education in addition to the questionnaire data (as explained below). Because the current study was a part of a larger research project, other measures were also included in the survey. Therefore, the present sample also partially overlaps with samples of other publications (Sindermann et al., 2020, 2021a,b; Rozgonjuk et al., 2021).

The online study was approved by the local institutional ethics committee of Ulm University, Ulm, Germany.

### Sample

A total of 421 individuals took part in the study. However, *n* = 4 were excluded because they reported not owning a smartphone; hence, the questionnaire for measuring levels of SmUD was not presented to them. Two additional participants replied with the same response option across all items in all main questionnaires (described in the following section “Measures”); finally, one participant had to be excluded because of not being eligible for the study (under 12 years old). Among the remaining participants, there were no missing data. The final sample comprised *N* = 414 (age range = 12–77, *M* = 33.64, *SD* = 13.49) participants. 151 (36.5%) were men and 263 (63.5%) were women. The vast majority of participants were from Germany (348; 84.1%), 53 participants (12.8%) were from Austria, and 13 individuals (3.1%) were from Switzerland. Almost half (192) of the participants (46.4%) held a higher educational degree, i.e., *n* = 147, over one-third (35.5%), had a University degree (*Hochschulabschluss*) and *n* = 45 (10.9%) a University of applied sciences degree (*Fachhochschule*). Approximately, one-quarter (24.4%), or *n* = 101 participants, had passed the A-levels (*Abitur*); *n* = 24 (5.8%) had a vocational diploma (*Fachabitur*). *N* = 73 (17.6%) participants had a secondary school leaving certificate (*Mittlere Reife*), *n* = 17 (4.1%) had a streamed secondary school degree (*Volks-/Hauptschulabschluss*), and *n* = 7 (1.7%) had not graduated.

### Measures

#### Short Version of the Smartphone Addiction Scale

We used the German translation [*d*eutsche *K*urz*v*ersion der *S*martphone*s*ucht*s*kala: d-KV-SSS as in Montag (2018)] of the *s*hort *v*ersion of the *S*martphone *A*ddiction *S*cale (SAS-SV) by Kwon et al. (2013). It reflects the degree of smartphone use disorder (SmUD) tendencies, including social and health impairment, and preoccupation. On this 10-item questionnaire, in which items are answered on a 6-point Likert scale (1 = strongly disagree to 6 = strongly agree), higher values in the range from 10 to 60 indicate a higher propensity towards SmUD. Example items comprise “The people around me tell me that I use my Smartphone too much,” “I have my Smartphone in my mind even when I am not using it,” and “I use my Smartphone longer than I had intended.” Internal consistency represented by Cronbach's α was 0.85 in the present sample.

#### The Big Five Inventory

Participants completed the German version of the Big Five Inventory (BFI; Rammstedt and Danner, 2017) consisting of 45 items answered on a 5-point Likert scale (1 = very inapplicable to 5 = very applicable). For the sake of completeness, we report internal consistencies and descriptive statistics of all Big Five variables, although, in our main analyses of the hypotheses, we only investigate the two personality traits, *n*eu*r*oticism (NR) and *co*nscientiousness (CO). Cronbach's alphas for NR and CO were α(NR) = 0.85 and α(CO) = 0.85. For *ex*traversion (EX), *op*enness (OP), and *ag*reeableness (AG), Cronbach's alphas were α(EX) = 0.88, α(OP) = 0.80, and α(AG) = 0.74.

#### Fear of Missing Out Scale

We assessed the degree of FoMO with the German version of the 10-item questionnaire of Przybylski et al. (2013) as provided in Spitzer (2015). This questionnaire uses a 5-point Likert scale (1 = not at all true of me to 5 = extremely true of me). Example items include “I fear others have more rewarding experiences than me,” “I get anxious when I don't know what my friends are up to,” and “It bothers me when I miss an opportunity to meet up with friends.” Internal consistency assessed by Cronbach's α was 0.75.

#### Mind-Wandering Questionnaire

We used the MWQ by Mrazek et al. (2013b) in this study. It measures trait levels of mind-wandering—including aspects of inattention (specified by its items)—representing a person's characteristics rather than a momentary state or snapshot of thought drifting, e.g., task-unrelated thoughts during a laboratory test. The five-item questionnaire (using a six-point response scale, 1 = almost never to 6 = almost always) includes the items “I have difficulty maintaining focus on simple or repetitive work” and “I do things without paying full attention.” The German version of the questionnaire was translated by the authors' research team by means of independent forward and backward translations, including subsequent discussions as well as adjustments if necessary (please find the German translation in the Supplementary Table 1). Cronbach's α was 0.85.

### Statistical Analyses

The following analyses were conducted with SPSS (MAC-Version 26.0.0.0, IBM Corp, 2019): internal consistency analyses (Cronbach's alphas), descriptive statistics, and mean-value comparisons with t-tests (Welch's t-tests were applied whenever necessary) to examine gender differences, Pearson's zero-order correlations, and Pearson's partial correlation analyses for measuring associations between variables in the focus of the present study.

Moreover, we conducted *c*onfirmatory *f*actor *a*nalyses (CFAs) to test the fit of the proposed factorial structure for each questionnaire using the *cfa()* function of the *lavaan* package (Rosseel, 2012). After controlling the model fit of each questionnaire, we computed a *s*tructural *r*egression *m*odel (SRM) as depicted in Figure 1, including all items using the *sem()* function of the *lavaan* package. For both CFA and SRM, we used the *d*iagonally *w*eighted *l*east *s*quare (DWLS)- and the *w*eighted *l*east *s*quares *m*ean and *v*ariance (WLSMV)-adjusted estimators as they deliver more accurate parameter estimates and a more robust model fit to the type of variables and non-normality compared to the *m*aximum-*l*ikelihood (ML) methodology (Mîndrilă, 2010).

In the structural regression model depicted in Figure 1, we controlled for gender in the personality traits (NR and CO) and for age in all key variables (NR, CO, FoMO, MWQ, SAS-SV). FoMO and mind-wandering were specified to mediate the relationships between the two personality variables (NR and CO) and SmUD (the outcome variable). Moreover, both the personality traits (NR, CO) and the mediators (FoMO, MW) were each allowed to correlate with each other, to be exploratively estimated by SRM (i.e., a covariance estimate of zero would also be permissible). All constructs were modeled as latent variables.

Model fit for each CFA and the SRM was evaluated with the standard indices considering their recommended thresholds for a good fit (Hu and Bentler, 1999; Hooper et al., 2008). For each construct, we report the following absolute fit indices: chi-square value including *p*-value and *d*egrees of *f*reedom (df), the root mean square error of approximation (good model fit assumed at RMSEA < 0.06) favoring models with fewer parameters, and the standardized root mean square residual (SRMR < 0.08) suitable for constructs in one model that differ in the length of the Likert-scale ranges of the items. Incremental fit indices are also stated, such as the Tucker–Lewis Index (TLI ≥ 0.95), preferring simpler models and being sensitive to small sample sizes, and the comparative fit index (CFI ≥ 0.95) well known for being less affected by sample size. The *cfa()* and *sem()* analyses were conducted using the statistical software R (R Core Team, 2020), version 4.0.2, with the graphical user interface RStudio (by RStudio Team, 2020), version 1.3.959.

To estimate an adequate sample size for SRM, we had a look at other studies that conducted similarly complex SEMs and executed several kinds of power analysis for SEM [e.g., semPOWER^8^ from Moshagen and Erdfelder (2016)]. With a hypothetical RMSEA value of 0.029 (indicating an excellent fit; Hooper et al., 2008), and the following parameters of a desired power of 0.99, an alpha of 0.01, *df* = 800 with 44 manifest variables, a sample size of *N* = 309 participants would be required. It has been suggested that using SEM, a sample size of at least *N* = 200 is recommended (Haenlein and Kaplan, 2004). In a similarly complex study with five latent variables, the sample analyzed contained approximately 400 participants (Balta et al., 2020); therefore, the sample size used in the current study is sufficient.

## Results

### Descriptive Statistics and Gender Differences

In Table 1, we report descriptive statistics for all key variables. In addition to the total sample, we included the mean and standard deviations for both male and female subsamples. Supplementary Table 2 shows the mean-value comparisons of the variables (Age, SAS-SV, FoMO, MWQ, BFI-NR, BFI-CO) between men and women. Only for the Big Five variables, including NR [*t*(412) = −2.56, *p* = 0.011) and CO [*t*(412) = −3.13, *p* = 0.002], gender differences occurred on a significant level, which is why gender is included in the SRM to influence NR and CO.

**Table 1:** Descriptive statistics.

	**Total sample (** ***N*** **=** **414)**						***n*** **(female)** **=** **263**		***n*** **(male)** **=** **151**	
**Variable**	***M***	***SD***	**Min**	**Max**	**Skewness**	**Kurtosis**	***M***	***SD***	***M***	***SD***
Age	33.64	13.49	12	77	0.625	−0.407	32.83	12.94	35.07	14.33
SAS-SV	28.74	9.49	10	54	0.120	−0.590	28.96	9.35	28.36	9.74
FoMO	2.41	0.61	1	4.1	0.336	−0.010	2.44	0.64	2.35	0.55
MWQ	3.24	0.95	1	6	0.202	−0.224	3.24	0.97	3.25	0.92
BFI-NR	2.99	0.78	1	4.75	−0.044	−0.599	3.07	0.78	2.87	0.77
BFI-CO	3.51	0.72	1.33	5	−0.316	−0.217	3.59	0.72	3.37	0.71
BFI-EX	3.39	0.84	1.25	5	−0.245	−0.698	3.48	0.82	3.24	0.86
BFI-OP	3.56	0.62	1.7	4.9	−0.354	0.082	3.62	0.63	3.47	0.61
BFI-AG	3.58	0.55	1.8	4.8	−0.347	−0.125	3.64	0.53	3.47	0.57

*SAS-SV, sum score of the short version of the Smartphone Addiction Scale (SAS-SV assessing SmUD); FoMO, average score of the Fear of Missing Out items; MWQ, average score of the Mind-Wandering Questionnaire items; Scales of the Big Five Inventory: BFI-NR, Neuroticism; BFI-CO, Conscientiousness; BFI-EX, Extraversion; BFI-OP, Openness; BFI-AG, Agreeableness*.

### Correlations Between Primary Variables

Table 2 shows zero-order bivariate Pearson's correlation coefficients for key variables (BFI-NR, BFI-CO, FoMO, MWQ, SAS-SV) of this study as well as associations with age and partial Pearson's correlations controlling for age. The significance of all zero-order bivariate correlations between the key variables compared to partial Pearson's correlations remained robust, i.e., were roughly of similar size and similarly significant. SmUD (SAS-SV) showed the strongest association with MWQ: *r* = 0.611, *p* < 0.001, *r*_p_(controlled for age) = 0.577, *p* < 0.001. As can be seen in Table 2, BFI-NR, FoMO, MWQ, and SAS-SV were positively linked.

**Table 2:** Zero-order bivariate Pearson's and partial Pearson's correlation coefficients representing associations between age and primary study variables among each other (*N* = 414).

**Variable**	**Age**	**SAS-SV**	**FoMO**	**MWQ**	**BFI-NR**	**BFI-CO**
SAS-SV	−0.306	1	0.323	0.577	0.350	−0.362
FoMO	−0.394	0.403	1	0.372	0.328	−0.193
MWQ	−0.266	0.611	0.434	1	0.367	−0.510
BFI-NR	−0.164	0.379	0.362	0.393	1	−0.274
BFI-CO	0.221	−0.404	−0.260	−0.538	−0.300	1

*SAS-SV, sum score of the short version of Smartphone Addiction Scale (SAS-SV assessing SmUD); FoMO, average score of the Fear of Missing Out items; MWQ, average score of the Mind-Wandering Questionnaire items; Scales of the Big Five Inventory: BFI-NR, Neuroticism; BFI-CO, Conscientiousness. Lower triangle: zero-order bivariate Pearson's correlation coefficients; upper triangle: partial Pearson's correlation coefficients controlled for age with df = 411. All bivariate correlations are significant at p < 0.001*.

Age and BFI-CO correlated positively. Both are negatively associated with the remaining key variables: BFI-NR, FoMO, MWQ, and SAS-SV. Correlations computed by gender (presented in Supplementary Tables 3, 4) support these findings, except for the partial Pearson's correlation between BFI-CO and FoMO in the male subsample (*r*_p_ = −0.086, *p* = 0.295), which is negative, but not significant. This suggests that the association of FoMO with BFI-CO in the whole sample (Table 2) is driven by the female subsample (*r*_p_ = −0.280, *p* < 0.001). Based on these results, we included the covariate age for all latent key constructs (BFI-NR, BFI-CO, FoMO, MWQ, SAS-SV) in the SRM (Figure 1).

### Confirmatory Factor Analyses and Structural Regression Model

First, we investigated the model fit of measurement models for each questionnaire with a series of CFAs. The results and the model fit improvement procedure of the FoMO construct are described in Supplementary Table 5.

**Figure 2:**
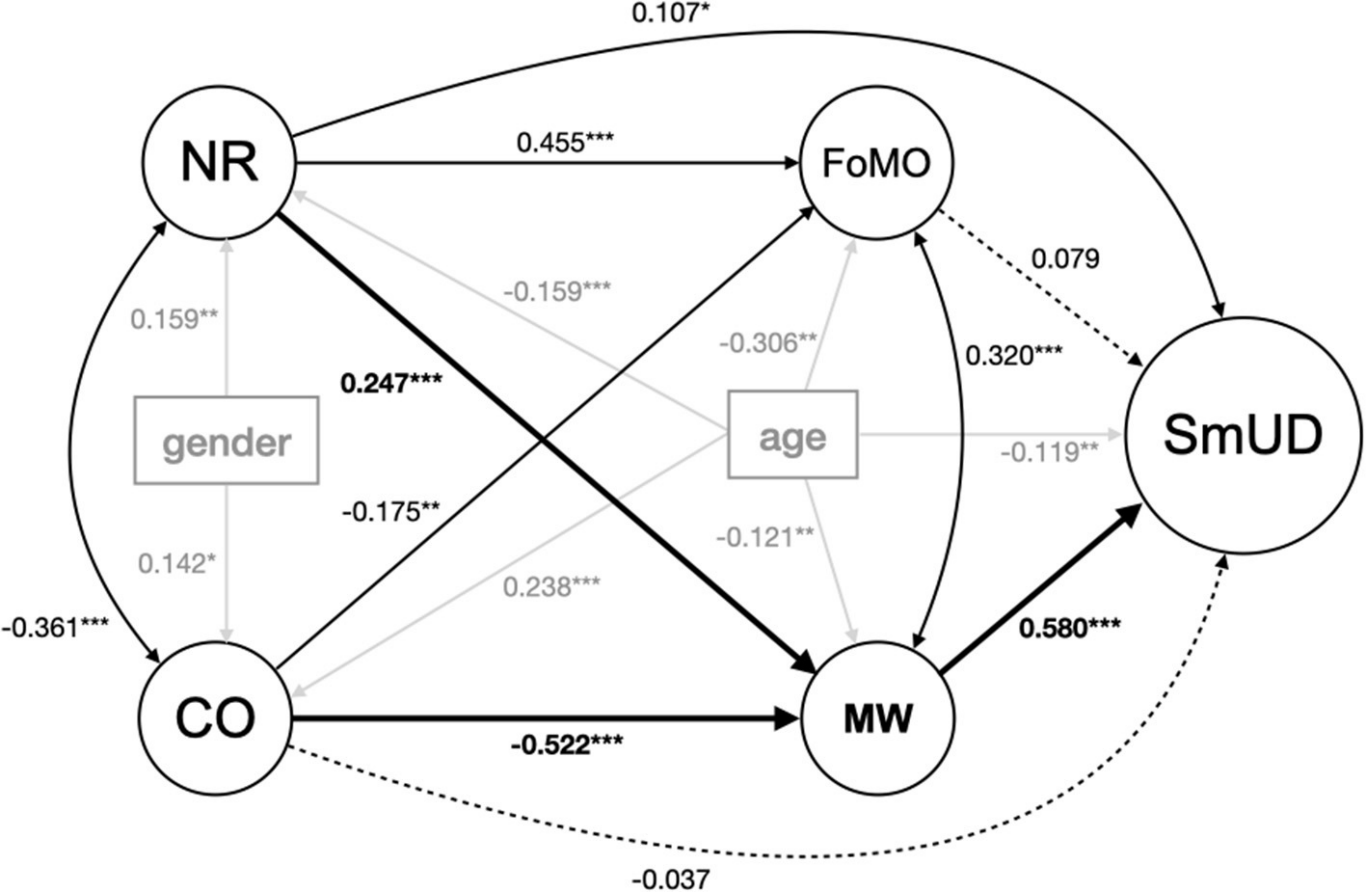
Results of the structural regression model displaying the standardized path coefficients. The graphical representation of the model displays latent constructs as circles (NR, CO, FoMO, MW, SmUD) and measured covariates (age, gender) as rectangles (manifest variables). Paths illustrated with one arrow tip signify a regression on the construct they point at, and two arrow tips indicate a covariance between two constructs. For reasons of clarity, items of the latent variables are not presented here, but were used to calculate the model. CO, Conscientiousness; NR, Neuroticism; MW, Mind-Wandering; FoMO, Fear of Missing Out; SmUD, Smartphone Use Disorder. *p < 0.05, **p < 0.01, ***p < 0.001. Dashed lines mark non-significant paths, and bold lines illustrate the dominating mediation pathways. Gray lines allow a better focus on the main associations investigated.

Table 3 shows the computed standardized path coefficients of the SRM (second last column), and partial Pearson's correlation coefficients (last column) for the total sample (*N* = 414). The effects of the two observed variables, age and gender, remained stable in the structural regression model compared with their corresponding bivariate correlations with all constructs of interest: NR, CO, FoMO, MWQ, and SmUD. Conscientiousness increases with age, whereas younger individuals have higher neuroticism. Both personality traits (neuroticism and conscientiousness) were predominantly found higher in women compared to men. Higher levels of FoMO, mind-wandering, and SmUD were more pronounced in younger people. The direct path coefficients computed in the structural regression model (Table 3) provided coherent results compared to bivariate correlation coefficients (Tables 2, 3). SmUD was positively linked to neuroticism (c1) and mind-wandering (b2). FoMO and mind-wandering were positively correlated with neuroticism (a1 and a2) and negatively linked to conscientiousness (a3 and a4). However, the direct effects of conscientiousness (c2) and FoMO (b1) on SmUD (gray shaded in Table 3 and displayed as dashed lines in Figure 2) were non-significant in the structural regression model. These links were completely mediated by mind-wandering: CO-MW-SmUD (a4·b2; illustrated as bold lines in Figure 2) and possibly also via the indirect path CO-FoMO-MW-SmUD, derived from the association between NR and CO. The direct effects of neuroticism (c1) and mind-wandering (b2) on SmUD were significant. This said, the effect size of c1-path (NR-SmUD) was reduced to one-third (*p*_SmUD,NR_ = 0.107, *p* = 0.037) compared to the corresponding bivariate partial correlation coefficient (*r* = 0.350, *p* < 0.001). Thus, the direct association between neuroticism (c1) and SmUD is partially mediated by the indirect pathway, including mind-wandering: NR-MW-SmUD (a2·b2) illustrated as bold lines in Figure 2. Please note that the present work is of correlational nature; wording implies no causality.

**Table 3:** Standardized path coefficients of the structural regression model compared to the bivariate correlation coefficients.

**Relationship of variables**					**Structural regression**	**Correlations**
**Direct effects** **(regressions)**			**Path**	***z*** **-value**	**Standardized path coefficients (SRM)**	**Partial Pearson's correlation coefficients**
**NR**	→	**SmUD**	**c1**	2.090	0.107*	0.350^***^
**CO**	→		c2	−0.714	−0.037	−0.362^***^
**FoMO**	→		b1	1.180	0.079	0.323^***^
**MW**	→		**b2**	9.188	0.580^***^	0.577^***^
**NR**	→	FoMO	a1	7.869	0.455^***^	0.328^***^
**CO**	→		a3	−3.227	−0.175^**^	−0.193^***^
**NR**	→	**MW**	a2	5.692	0.247^***^	0.367^***^
**CO**	→		**a4**	−11.372	–0.522^***^	−0.510^***^
**Gender**	→	NR	–	2.933	0.159^**^	0.114^*^
		CO	–	2.565	0.142^*^	0.175^***^
		SmUD	–	−2.830	−0.119^**^	–0.306^***^
		FoMO	–	−6.818	−0.306^***^	–0.394^***^
**Age** ^a^	→	MW	–	−3.104	−0.121^**^	–0.266^***^
		NR	–	−3.420	−0.159^***^	–0.164^***^
		CO	–	5.137	0.238^***^	0.221^***^
**Indirect effects (mediation)**						
**NR** →	FoMO →	**SmUD**	a1·b1	1.183	0.036	–
**CO** →			a3·b1	−1.110	−0.014	–
**NR** →	**MW** **→**		**a2·b2**	5.032	0.143^***^	–
**CO** →			**a4·b2**	−7.277	–0.303^***^	–
**Total effects (direct** **+** **indirect)**						
**NR** →	SmUD	c1 + (a1·b1) + (a2·b2)		6.133	0.286^***^	–
**CO** →		c2 + (a3·b1) + (a4·b2)		−7.686	−0.354^***^	–
**(Non-)Covariances**						
**NR**		CO		−6.670	−0.361^***^	−0.274^***^
**FoMO (latent)**		MW (latent)		4.432	0.320^***^	0.372^***^
**FoMO (item 1)**		FoMO (item 2)		7.197	0.665^***^	–

*In the second last column, both latent (calculated from item information) and observed variables are standardized, except for gender. Last column: Equivalent partial Pearson's correlation coefficients for all key variables (gray upper triangle of Table 2) and gender, as well as Pearson's correlation coefficients of age with all key variables (second column in Table 2); gender: male, 0; female, 1; Bold type depicts mind-wandering paths, Highlighted in gray displays the major differences between bivariate correlations and SRM-path coefficients*,

* *p < 0.05*,

** *p < 0.01*,

*** *p < 0.001;*

a *Correlations with age are not partial*.

## Discussion and Conclusions

In the present study, we investigated the indirect effects of FoMO and mind-wandering in the relations between SmUD and the personality traits, NR and CO.

As theoretical basis, we combined Billieux's pathways model (2012) and the I-PACE model of Brand et al. (2016, 2019) in the context of the taxonomy model from Montag et al. (2021). According to the I-PACE model, SmUD might be highly influenced by personal characteristics as well as other factors interacting with these variables. In the current work, personality traits (specifically, neuroticism and conscientiousness) can be understood as personal characteristics according to the I-PACE model, while mind-wandering and FoMO could be affective and cognitive variables. Our aim was to better understand relevant processes underlying the still novel phenomenon of SmUD. We investigated if neuroticism and conscientiousness predict SmUD, and whether mind-wandering and FoMO would mediate the link between personality and SmUD.

Importantly, by using an SEM approach, we also addressed the limitations raised by Brand et al. (2016), namely, that isolated research of a few variables simplifies the nature of complex constructs, such as SmUD, to a large extent.

First, we hypothesized that “Higher neuroticism and lower conscientiousness are associated with higher SmUD” (H1); “Higher neuroticism is associated with higher FoMO and higher mind-wandering levels” (H2); and “Lower conscientiousness is associated with higher FoMO and higher mind-wandering levels” (H3). These hypotheses found support from the data in bivariate analyses. The results of Pearson's correlation analyses, both with and without controlling for age, showed that higher neuroticism and lower conscientiousness were linked to higher levels of SmUD, consistent with previous findings (Marengo et al., 2020). In a multivariate structural regression model (results presented in Table 3), neuroticism was still positively linked to SmUD, but the direct CO-SmUD link was not significant; instead, it was fully mediated by mind-wandering.

All pathways of the SRM depicted in Figure 1 were significant in bivariate correlation analyses, aligning with findings in previous studies. Mind-wandering showed the strongest significant associations with all other key variables. Hence, it is not surprising that it was a significant factor in both mediation pathways (in line with hypothesis H4: “The associations of neuroticism and conscientiousness with SmUD are mediated by FoMO and mind-wandering”). To our knowledge, this is the first study exploring a link between the two Big Five personality traits (neuroticism and conscientiousness) as well as mind-wandering and SmUD in a multivariate model. A person with high neuroticism, who is more worrisome, temperamental, and moody, may be more inclined to being inattentive. Moreover, self-disciplined, efficient, and conscientious individuals probably may be less prone to drift away with their thoughts as they are attributed to be focused (McCrae and John, 1992). These interrelations are reflected by our data in both bi- and multivariate analyses. The positive association between mind-wandering and SmUD was replicated compared to previous research (Zheng et al., 2014; Hadar et al., 2017; Marty-Dugas et al., 2018; Kim et al., 2020). Additionally, the links of higher FoMO between both higher neuroticism and lower conscientiousness were also reproved (Stead and Bibby, 2017). Higher levels of FoMO, though, only denote a strong significant correlation with higher SmUD levels in the isolated bivariate analyses, which is in line with the literature (Chotpitayasunondh and Douglas, 2016; Elhai et al., 2016, 2018, 2020c; Oberst et al., 2017; Gezgin, 2018; Wolniewicz et al., 2018). However, the results of the SRM showed that this direct relationship (FoMO-SmUD) was not significant in the multivariate analysis (SRM). This finding suggests that the fourth hypothesis (H4) of the present study only finds partial support from the data, as mediation between personality traits and SmUD both times involves mind-wandering as a mainstay.

The results showed that mind-wandering may be a central factor in the personality–SmUD association. This is in line with the I-PACE model, according to which mind-wandering might function as an affective and/or cognitive variable mediating the relation between personality traits and SmUD. Interestingly, FoMO's potential effects were low in the presence of mind-wandering. This might have its origin in the related nature of both constructs, reflected also in the significant covariance of FoMO and mind-wandering (Table 3). People with a scattered frequently drifting mind (mind-wandering) might be more familiar with the idea of what their peers are doing in the meantime (FoMO), and vice versa. However, both pairs—neuroticism and FoMO, and mind-wandering and conscientiousness—show stronger associations towards the other mediation candidate [Figure 2: NR-FoMO (stronger) vs. NR-MW (weaker) and CO-MW (stronger) vs. CO-FoMO (weaker)], respectively. This might account for the overlap of the concepts, FoMO and mind-wandering. For construct overlaps on item level, see page 5 of the Supplementary Material.

### The Two-Fold Nature of Mind-Wandering Triggered by Smartphone Notifications

Next to its stable tendency (trait), state levels of mind-wandering can also be measured by capturing momentary task-unrelated thoughts. As such, smartphone-induced prompts can stimulate a mind to wander, which might ultimately lead to an increased overall frequency of mind-wandering episodes. Especially, individuals with higher tendencies towards SmUD might be more vulnerable to such smartphone-induced stimuli. In this context, state mind-wandering describes interruptions by thoughts unrelated to the task on which one is currently focusing (Smallwood and Schooler, 2006). On the contrary, state mind-wandering might also influence smartphone use: When your mind wanders, you might to think more about your smartphone and, finally has a look at it, which may ultimately lead to SmUD. Therefore, inattentive behavior (state mind-wandering) and problematic smartphone use (Nixon, 2017) might be mutually dependent, described as follows.

Research from related fields shows that push notifications announced by acoustic and/or visual alerts on the smartphone lead to numerous daily interruptions resulting in performance drain (Stothart et al., 2015). Even the sole presence of a smartphone interferes negatively with cognitive capacity (Ward et al., 2017). A study with a 2-week longitudinal design (self-reports accompanied by experiments) underpins that interruptions caused by smartphone notifications increase state mind-wandering in form of inattention^9^ (Kushlev et al., 2016). Owning a mobile phone or the duration of consuming entertainment on a mobile phone has been discovered to be significantly linked to inattention in a sample of Chinese adolescents (Zheng et al., 2014). Moreover, in a Hebrew population, heavy smartphone use, in particular, the frequency of smartphone usage, predicted the degree of impaired attention (Hadar et al., 2017).

**Figure 3:**
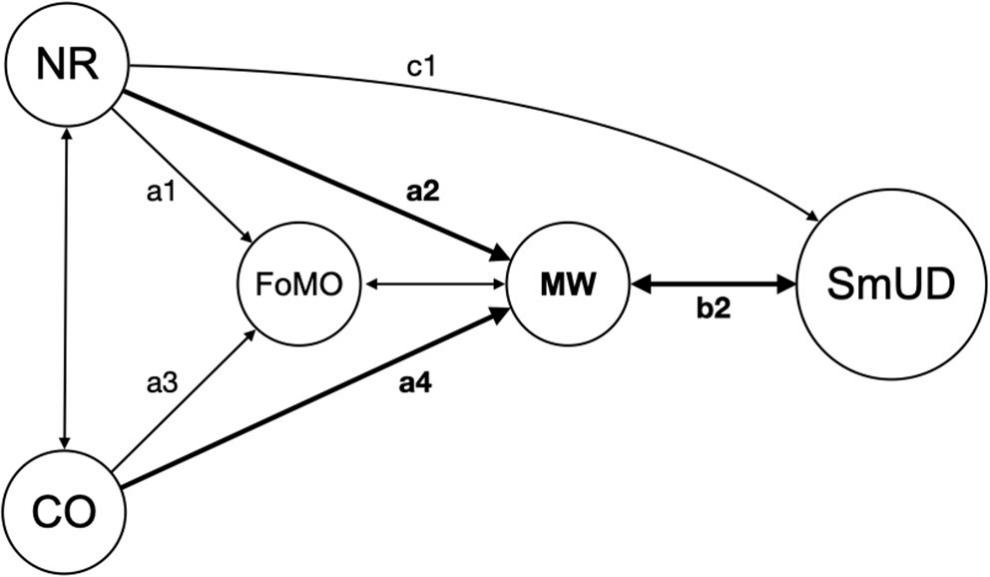
Illustration of the significant associations between the key variables calculated by SRM including the interrelation (b2) between MW and SmUD, reinforcing each other; CO, Conscientiousness; NR, Neuroticism; MW, Mind-Wandering; FoMO, Fear of Missing Out; SmUD, Smartphone Use Disorder.

These studies indicate that mind-wandering might also be triggered by the use or the sole presence of the smartphone in form of frequent disturbances, which reduce attention, thus hindering the performance of ongoing tasks. Experiments in these settings indicate that people compensate those externally induced interruptions and, contrary to expectations, complete the task they are focused on even quicker and with the same quality (Zijlstra et al., 1999; Mark et al., 2008). However, the downside of this is disrupted emotion regulation, which may lead to decreased well-being (for instance, higher levels of stress, feelings of frustration and exhaustion). The evolving unpleasant feelings from the latter in turn create an urge for instant gratification and/or compensation, allowing maladaptive coping mechanisms to kick in. These may result in further active smartphone use, forming a vicious circle (Kushlev et al., 2016) between a “wandering mind” and smartphone-induced interruptions (Figure 3: b2). Wilmer et al. (2017) gave an overview about the connection between habitual smartphone use and cognition focusing on effects on memory, gratification, and attention in their review. Related to this, Liebherr et al. (2020) build a first hypothetical model about immediate- and long-term effects of smartphone use on the triad of attention, working memory, and inhibition. Due to the close direct connection of mind-wandering and SmUD, the assumption is obvious that individuals with an innately wandering mind (trait) may be even more prone to become trapped in the mutually reinforcing interrelationship of mind-wandering and SmUD.

This said, a wandering mind can also be seen in a positive light. For instance, the right dose of mind-wandering might foster creativity (Baird et al., 2012); however, it should be noted that the kind of mind-wandering in Baird et al. (2012) was not assessed in the present study. Rather, the focus was on the dark side of mind-wandering, such as drifting thoughts or inattention (again, more in the sense of a trait).

### Interventions Targeting Dysfunctional Mind-Wandering

The central role of mind-wandering between the predominant personality factors and SmUD is more clearly illustrated in Figure 3. The results of the present study suggest that mind-wandering might be a promising target to reduce SmUD and break the “vicious cycle” described in the section “The Two-Fold Nature of Mind-Wandering Triggered by Smartphone Notifications”. Mindfulness training has been identified to lower the occurrence of mind-wandering: A two-week course (Mrazek et al., 2013a) or eight minutes of mindful breathing (Mrazek et al., 2012) showed effect in reducing mind-wandering-related behaviors. Another helping intervention to decrease levels of mind-wandering and gain back concentration to work in flow is to batch smartphone notifications (Fitz et al., 2019).

Following the quote “Concentration of consciousness, and concentration of movements, diffusion of ideas and diffusion of movements go together.” from Ribot (1890, p. 24), Carriere et al. (2013) investigated mind-wandering and fidgeting as this specific form of body movement that turned out to be linked to attention. In individuals whose minds spontaneously wander, a hand or other parts of the body tend to wander or move as well. An example of a tool that was designed especially for people who frequently fidget is the “fidget cube”^10^. Further investigation of intervention effects on mind-wandering could encompass to what extent the aforementioned game device or similar tools improve the attention capacity. The two mutually reinforcing variables and the therapeutic effect of reducing mind-wandering on SmUD should be explored in depth in future studies. This could increase productivity and mental well-being of people who are prone to mind-wandering, i.e. who are also easily distracted by digital devices.

### Limitations

The following shortcomings of the present study are to be mentioned. First, the focus on the smartphone device itself in many works (including this one) may need a refocus, because users attach to applications running on the smartphone but not necessarily to the smartphone itself (Pontes et al., 2015). Several recent empirical works showed a moderate to strong overlap between the overuse of smartphones and social media applications, which might induce *s*ocial *n*etwork-*u*se *d*isorders (SNUDs). Among others, excessive smartphone and WhatsApp use overlap very strongly [Sha et al., 2019; for shared variance with other platforms, see also Rozgonjuk et al. (2020)].

Second, the data were cross-sectional and although the structural regression model included directed pathways, the causal links stem from theory (assuming that personality is relatively stable over time and affects behavior) rather than an experimental study. Therefore, the causality should be interpreted with care.

Third, our study did not include objectively measured smartphone use. Although SmUD cannot be equated with the frequency and duration of smartphone use as posited in the I-PACE model (Brand et al., 2019), the inclusion of objectively measured data on smartphone use could provide further insight into the relationships between psychological variables and smartphone use. Recently, a novel field of research, *Psychoinformatics*, has emerged that could contribute additional insights to self-administered questionnaires, including how and when the device is used for what (Montag et al., 2014, 2015b). Furthermore, it is still under investigation whether FoMO (Wegmann et al., 2017; Balta et al., 2020) and mind-wandering are state or trait variables; however, states and traits are connected (please note that in Wegmann et al., 2017, trait- and state-FoMO are operationalized differently, i.e., state-FoMO stands for online FoMO). At least for the mind-wandering construct, trait/state-effects seem to validate each other (Seli et al., 2016). However, deliberate and spontaneous mind-wandering might need to be disentangled (Carriere et al., 2013; Seli et al., 2015, 2016; Vannucci and Chiorri, 2018). Moreover, even different forms of smartphone use (general and absent-minded^11^) have been considered by Marty-Dugas et al. (2018) in this context.

Please note that mind-wandering was assessed in the sense of a disposition towards inattentive states in the present study. Therefore, a wandering mind being even associated with creativity (e.g., Baird et al., 2012; Leszczynski et al., 2017) has not been covered in this work (but this might be an interesting lead for future studies in the context of smartphone use). Finally, due to the nature of our sample, as described in the section “Sample”, our results may only be partially generalizable to other populations.

### Conclusion

In sum, this is the first study that investigated the effects of personality, FoMO, and mind-wandering on SmUD. Most interestingly, mind-wandering, a variable barely examined in the context of SmUD so far, turned out to be an interesting new construct to explain SmUD. Based on that, new treatment opportunities for SmUD can arise. Future research should investigate the associations between mind-wandering and SmUD more in depth by implementing an experimental study design supported by psychoinformatic methodologies to examine causal relations. Overall, the findings from our study provide a starting point to work on interventions related to mind-wandering, such as mindfulness trainings, to reduce or even treat SmUD. This can significantly improve well-being and quality of life for many people.

## Data Availability Statement

The data will be made available upon scholarly request via the first author.

## Ethics Statement

The online study was approved by the Local Ethics Committee of Ulm University, Helmholtzstraße 20 (Oberer Eselsberg), 89081 Ulm, Germany, https://www.uni-ulm.de/en/einrichtungen/ethikkommission-der-universitaet-ulm/.

## Author Contributions

MM and CM planned the present study. CM collected the data. MM and CS created the research model and the SRM analysis. MM implemented statistical analysis, which was independently checked by CS and DR. Both reviewed the SRM and gave helpful feedback. MM wrote the present manuscript and interpreted the data. CS, DR, and CM critically revised the manuscript. All authors contributed to the article and approved the submitted version.

## Conflict of Interest

The authors declare that the research was conducted in the absence of any commercial or financial relationships that could be construed as a potential conflict of interest.

## Publisher's Note

All claims expressed in this article are solely those of the authors and do not necessarily represent those of their affiliated organizations, or those of the publisher, the editors and the reviewers. Any product that may be evaluated in this article, or claim that may be made by its manufacturer, is not guaranteed or endorsed by the publisher.

## Footnotes

^1^*I*maginal *P*rocess *I*nventory (IPI) for mind-wandering both the *D*ay*d*reaming *F*requency *S*cale (DDFS) (factor 3) and the mind-wandering subscale (factor 19).

^2^*S*ustained *A*ttention to *R*esponse *T*ask (SART).

^3^Questions considering mind-wandering: “I am daydreaming/my mind is wandering about things unrelated to the task,” “I am not very alert/my mind is blank”.

^4^*M*ind-*W*andering: *S*pontaneous (MW-S) and *M*ind-*W*andering: *D*eliberate (MW-D) scale.

^5^*M*indful *A*ttention *A*wareness *S*cale–*L*apses *O*nly (MAAS-LO), *A*ttention-*R*elated *C*ognitive *E*rrors *S*cale (ARCES), *M*ind-*W*andering: *S*pontaneous (MW-S) and *M*ind-*W*andering: *D*eliberate (MW-D) scale.

^6^Measured by the Korean version of the *A*dult *A*ttention *D*eficit *H*yperactivity *D*isorder *S*cale (AADHDS).

^7^Adult *A*DHD (Attention Deficit Hyperactivity Disorder) *S*elf-*R*eport *S*cale (ASRS-Part A).

^8^ https://sempower.shinyapps.io/sempower

^9^Measured by an *A*ttention *D*eficit *H*yperactivity *D*isorder (ADHD) test based on the criteria of the Diagnostic and Statistical Manual-V (DSM-V).

^10^Available online at: https://www.antsylabs.com/collections/fidget/products/fidget-cube.

^11^General smartphone use: frequency of texts sent and received, absent-minded smartphone use: usage without purpose.
