# Supplementary material for: Mind-Wandering Mediates the Associations Between Neuroticism and Conscientiousness, and Tendencies Towards Smartphone Use Disorder
Source: Front Psychol. 2021 Aug 30;12:661541. doi: 10.3389/fpsyg.2021.661541 (PMC8435592; doi:10.3389/fpsyg.2021.661541)
Supplement: Supplementary file 1 [file Data_Sheet_1.pdf]

Supplementary Material

1 Items of the Mind-Wandering Questionnaire (MWQ) translated into German

Supplementary Table 1 | German translation of the MWQ-items (Mrazek, Phillips, Franklin, Broadway, & Schooler, 2013)

	English items	German items
**1.**	I have difficulty maintaining focus on simple or repetitive work.	Ich habe Schwierigkeiten, mich auf einfache Arbeiten oder sich wiederholende Aufgaben zu konzentrieren.
**2.**	While reading, I find I haven't been thinking about the text and must therefore read it again.	Beim Lesen merke ich, dass ich mich nicht auf den Text konzentriert habe und ihn deswegen nochmal lesen muss.
**3.**	I do things without paying full attention.	Ich tue Dinge ohne voll bei der Sache zu sein.
**4.**	I find myself listening with one ear, thinking about something else at the same time.	Ich ertappe mich, dass ich mit einem Ohr zuhöre und zur selben Zeit an etwas anderes denke.
**5.**	I mind-wander during lectures or presentations.	Ich lasse meine Gedanken während Vorträgen oder Präsentationen schweifen.

The response items ranged from *1-almost never* to *6-almost always*, in German: *1* = *fast nie* to *6* = *fast immer* on a 6-point Likert scale.

2 Gender-specific investigation for all key variables (t-test)

We used independent t-tests to investigate gender differences concerning age and the primary variables (neuroticism (NR), conscientiousness (CO), Smartphone Addiction Scale (SAS-SV) assessing Smartphone Use Disorder (SmUD), Fear of Missing Out (FoMO) and MWQ) of the study, see Supplementary Table 2.

Supplementary Table 2 | t-test results to identify gender differences of age and the primary variables of the study (alternatively, Levene's test for variance equality)

	Levene's test		t-test		
**Variable**	**F**	***p***	**T**	***df***	***p* (2-tailed)**
Age	2.025	0.155	1.635	412	0.103
SAS-SV	0.142	0.706	-0.624	412	0.533
FoMO[a]	4.588	0.033	-1.622	352.19	0.106
MWQ	0.285	0.594	0.025	412	0.980
**BFI-NR**	**0.131**	**0.718**	**-2.563**	**412**	**0.011**
**BFI-CO**	**0.008**	**0.929**	**-3.133**	**412**	**0.002**

SAS-SV, sum score of the short version of the Smartphone Addiction Scale (SAS-SV assessing SmUD); FoMO, average score of Fear of Missing Out; MWQ, average score of Mind-Wandering Questionnaire; Scales of Big Five Inventory: BFI-NR, Neuroticism; BFI-CO, Conscientiousness. [a] *Welch's* t-test of FoMO in consideration of gender differences of variances as Levene's test is significant ($p = 0.033$)

3 Zero order Pearson's and partial Pearson's correlation coefficients for male and female subsamples

Correlations for males and females revealed the following results: All associations in both subsamples remain significant like in the whole sample, at a significance level of $p < 0.05$. The only exception is the partial Pearson correlation between CO and FoMO ($r = -0.086$, $p = 0.295$) in the male subsample. The aforementioned correlation of CO and FoMO in the whole sample ($r = -0.193$, $p < 0.001$) is driven by the female subsample ($r = -0.280$, $p < 0.001$) as displayed in Supplementary Table 4.

Supplementary Table 3 | Zero-order Pearson and partial correlation coefficients of variables of the male subsample (*n* = 151, *df* = 148)

Variable	Age	SAS-SV	FoMO	MWQ	BFI-NR	BFI-CO
**SAS-SV**	-0.428***	1	0.276***	0.565***	0.352***	-0.297***
**FoMO**	-0.338***	0.379***	1	0.200*	0.267***	**-0.086**
**MWQ**	-0.358***	0.630***	0.296***	1	0.242**	-0.485***
**BFI-NR**	-0.163*	0.384***	0.303***	0.281***	1	-0.162*
**BFI-CO**	0.387***	-0.413***	-0.205*	-0.556***	-0.211**	1

The bolt type depicts the non-significant value. SAS-SV, sum score of the short version of the Smartphone Addiction Scale (SAS-SV assessing SmUD); FoMO, average score of Fear of Missing Out; MWQ, average score of Mind-Wandering Questionnaire; Scales of Big Five Inventory: BFI-NR, Neuroticism; BFI-CO, Conscientiousness. Gray cells highlight Pearson's partial correlation coefficients controlled for age with $df = 148$, $*p < 0.05$, $**p < 0.01$, $***p < 0.001$.

Supplementary Table 4 | Zero-order Pearson and partial correlation coefficients of variables of the female subsample (*n* = 263, *df* = 260)

Variable	Age	SAS-SV	FoMO	MWQ	BFI-NR	BFI-CO
**SAS-SV**	-0.224***	1	0.364***	0.583***	0.354***	-0.396***
**FoMO**	-0.424***	0.416***	1	0.463***	0.357***	-0.280***
**MWQ**	-0.214***	0.603***	0.501***	1	0.440***	-0.525***
**BFI-NR**	-0.152*	0.375***	0.384***	0.458***	1	-0.376***
**BFI-CO**	0.141*	-0.414***	-0.311***	-0.538***	-0.389***	1

SAS-SV, sum score of the short version of the Smartphone Addiction Scale (SAS-SV assessing SmUD); FoMO, average score of Fear of Missing Out items; MWQ, average score of Mind-Wandering Questionnaire items; Scales of Big Five Inventory: BFI-NR, Neuroticism; BFI-CO, Conscientiousness. Gray cells highlight Pearson's partial correlation coefficients controlled for age with $df = 260$, $*p < 0.05$, $**p < 0.01$, $***p < 0.001$.

4 Model fit by Confirmatory Factor Analysis (CFA)

As can be seen in Supplementary Table 5, in general, the CFAs of the scales showed a good fit. However, the initial results of the uni-dimensional FoMO construct showed a rather mediocre fit (gray highlighted in Supplementary Table 5). A closer look into the CFA of the FoMO model itself revealed two very poor standardized factor loadings (< 0.300) for two items: Item 8 "When I have a good time it is important for me to share the details online (e. g. updating status)." and item 9 "When I miss out on a planned get-together it bothers me." Moreover, modification indices in the CFA of the initial FoMO model indicated a high relatedness of the following two items: Both, item 1 "I fear *others* have more rewarding experiences than me.", and item 2 "I fear *my friends* have more rewarding experiences than me." show a high degree of conceptual similarity. Therefore, we connected them by adding a residual covariance in the CFA of FoMO. A similar approach has been used elsewhere, among others in Rozgonjuk et al. (2019).

Supplementary Table 5 | Model fit indices for each scale (latent variables in CFA) and the structural regression model (SRM)

Variable	Chi^2	*df*	RMSEA	RMSEA [90% CI]	CFI	TLI	SRMR
**SAS-SV**	189.955	35	0.104	0.089 to 0.188	0.973	0.965	0.078
**FoMO**	529.046	35	0.185	0.171 to 0.199	0.874	0.838	0.141
**FoMO_mod**	54.389	19	0.067	0.047 to 0.088	0.990	0.985	0.056
**MWQ**	34.517	5	0.120	0.084 to 0.159	0.992	0.985	0.049
**NR**	141.860	20	0.121	0.103 to 0.141	0.974	0.964	0.070
**CO**	104.574	27	0.083	0.067 to 0.101	0.986	0.981	0.060
**SRM (FoMO_mod)**	2,383.538	803	0.069	0.066 to 0.072	0.962	0.961	0.071

Latent variables: SAS-SV, sum score of the short version of the Smartphone Addiction Scale (SAS-SV assessing SmUD); FoMO, average score of Fear of Missing Out items; MWQ, average score of Mind-Wandering Questionnaire items; Scales of Big Five Inventory: NR, Neuroticism; CO, Conscientiousness; FoMO_mod: modified construct of FoMO to assess model fit by omitting items 8 and 9 as well as covarying item 1 with item 2, SRM (FoMO_mod): complete SRM using the modified latent FoMO construct (omitting item 8 and 9, allowing residual covariance of item 1 and 2), used estimator in R: WLSMV, all p-values of Chi^2 were < 0.001.

5 Construct overlap on item level

On item level, modification indices (from CFA) displaying construct-overlapping relationships, revealed that the inverted item no. 43 ("I see myself as someone who is easily distracted.") of the conscientiousness factor in the Big Five additionally accounts for the latent Mind-Wandering (MW) construct (0.677). This might be a hint underpinning the full mediation between CO and SmUD (c1), which is superseded by the indirect mind-wandering pathway CO–MW–SmUD (a2 · b2) in the structural regression model.
In contrast, the direct association between NR and SmUD (c1 = 0.107, $p = 0.037$) faded in comparison to its bivariate relation of both constructs ($r = 0.350$, $p < 0.001$), as it is only partially mediated through mind-wandering (NR–MW–SmUD: a2 · b2 = 0.143, $p < 0.001$). See Table 3, Figure 1 and 2 in the main manuscript for illustration.

On a side note, item no. 43 of the CO-construct also overlaps with the following latent constructs:

- SmUD (0.510),
- FoMO (0.421) and
- moderately with NR (0.360)

The overlapping character only of CO item no. 43 could be one driver for the strong mediation pathway CO–MW–SmUD.

References

Mrazek, M. D., Phillips, D. T., Franklin, M. S., Broadway, J. M., & Schooler, J. W. (2013). Young and restless: Validation of the Mind-Wandering Questionnaire (MWQ) reveals disruptive impact of mind-wandering for youth. *Frontiers in Psychology*, *4*(AUG), 1–7. https://doi.org/10.3389/fpsyg.2013.00560

Rozgonjuk, D., Elhai, J. D., Ryan, T., & Scott, G. G. (2019). Fear of missing out is associated with disrupted activities from receiving smartphone notifications and surface learning in college students. *Computers and Education*, *140*(May), 103590. https://doi.org/10.1016/j.compedu.20190.05.016
